# Antero-posterior lingual sliding retraction system for orthodontic correction of hyperdivergent Class II protrusion

**DOI:** 10.1186/1746-160X-10-22

**Published:** 2014-06-05

**Authors:** Soon-Yong Kwon, Hyo-Won Ahn, Seong-Hun Kim, Young-Guk Park, Kyu-Rhim Chung, Cheol-Ho Paik, Gerald Nelson

**Affiliations:** 1 Department of Orthodontics, School of Dentistry, Kyung Hee University, #1 Hoegi-dong, Dongdaemun-gu, Seoul 130-701, South Korea; 2 Department of Orthodontics, School of Medicine, Ajou University, Suwon, South Korea; 3 Department of Orthodontics, Seoul National University, Seoul, South Korea; 4 Division of Orthodontics, Department of Orofacial Science, University of California San Francisco, San Francisco, CA, USA

## Abstract

**Background:**

This report introduces a lingual bonded retraction system (Kinematics of Lingual Bar on Non-Paralleling Technique, KILBON) for efficient sliding mechanics combined with vertical control of the anterior and posterior teeth, which is suitable for Class II hyperdivergent patients.

**Methods:**

Design and biomechanics of the KILBON System were described. Two adults with hyperdivergent class II malocclusion were treated with the KILBON system and temporary skeletal anchorage devices (TSADs) on the palate. The first patient was treated with conventional KILBON system on the upper arch and detailed with lingual appliances. The second patient showed the modified design of the KILBON when applied to a low palatal vault.

**Results:**

A large amount of intrusion and retraction of the anterior teeth and simultaneous intrusion of the posterior segment were achieved in short treatment time. Concomitant counterclockwise rotation of the mandible improved the esthetic profile. Periodontal support without dehiscence or bone loss was confirmed on anterior region in spite of large amount of retraction.

**Conclusions:**

This report presented a lingual retraction system that provides simple and effective vertical and sagittal control of both anterior and posterior teeth. The biomechanics are dependable for correcting a dentoalveolar protrusion in a patient with Class II hyperdivergent skeletal pattern.

## Background

As esthetic concerns have increased in orthodontic fields, lingual appliances have become more sophisticated. Biomechanics for lingual orthodontics are not the same as for labial techniques [1,2]. The most clinically challenging problem is torque control of the maxillary incisors during retraction [1,3].

Several studies have introduced incisor retraction using lever arms combined with TSADs [4,5]. However, when using TSADs combined with conventional lingual bonded appliances, unwanted side effects such as distal tipping of the posterior teeth or round tripping of anterior teeth are frequently occurred due to the friction [6].

**Figure 1:**
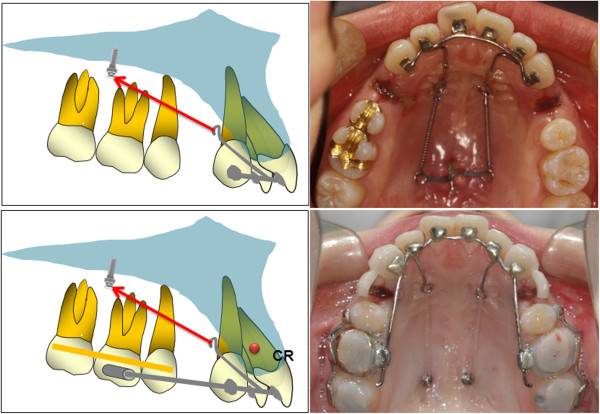
Anterior segmental retraction using lingual appliances: the lingual retractor (upper row) and the KILBON system (lower row).

The lingual retractor which splints the anterior segment into one unit can minimize or eliminate the effect of slot play, the need to round-trip the anterior teeth, and can shorten the treatment time [7]. Posterior teeth usually have no bonded attachments and maintain their original position [8].Unfortunately, current treatment protocols for lingual appliances cannot produce dependable vertical or sagittal control of both the anterior and posterior segment during retraction. The aim of this report is to present a new design of an antero-posterior lingual sliding retraction system (Kinematics of Lingual Bar on Non-Paralleling Technique, KILBON) and its application for the treatment of class II hyperdivergent patients (Figure 1).

## Methods

### Design and biomechanics

**Figure 2:**
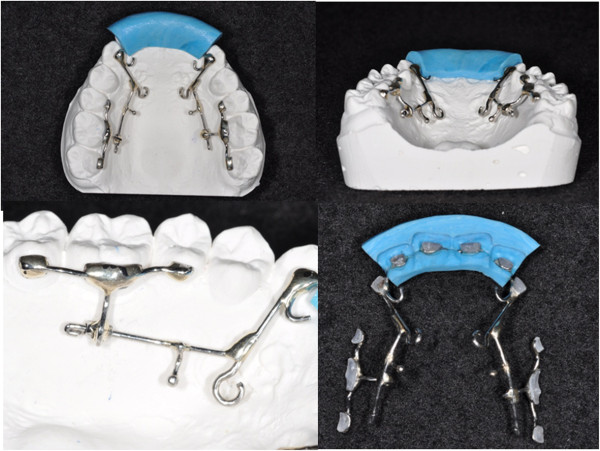
Design of the KILBON system and its transfer jig.

The KILBON system, as a customized lingual appliance, consists of two parts (Figure 2). One is the anterior segment made of a 0.036-inch stainless-steel wire soldered to mesh pads that splints the six anterior teeth into a single unit. Two long lever arms are soldered to the anterior segment, designed to direct the vector of the retraction force through the center of resistance. The hooks of the arms are approximately 20 mm apically from the base arch. The posterior segments are splinted together into one unit with a soldered extension arm ending in a short tube (diameter 1 mm). The tube aperture is parallel to the occlusal plane and functions as sliding yoke. A 0.036 SS guide wire is soldered to the retraction hooks and extends distally through the tube. The play between the posterior extension wire and the tube is 0.1 mm. The posterior extension wire gives vertical stabilization to the anterior group of teeth, preventing unwanted extrusion or intrusion side effects. Usually, one or two TSAD are applied to paramedian area of the palate (Jin-biomed co., Bucheon, Korea). Depending on the combinations of the position of the lever arm and placement of the TSADs, anterior teeth can be intruded with controlled tipping or bodily movement. Typically, the amount of intrusion of the posterior teeth is less than the anterior teeth, which results in flattening of the occlusal plane.

### Application procedure

**Figure 3:**
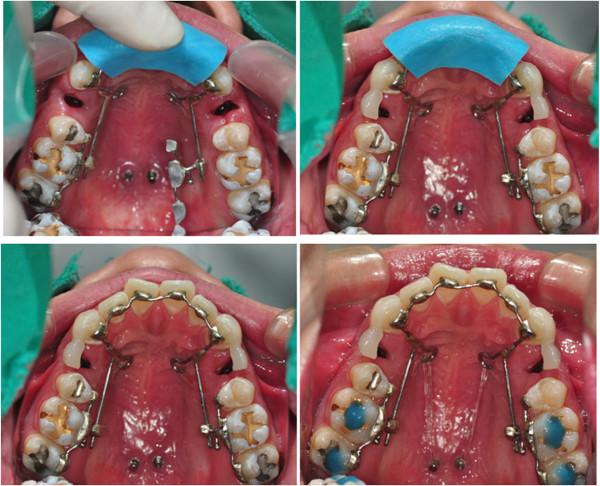
Bonding procedures of the KILBON system.

An anterior transfer jig that covers the four maxillary anterior teeth is fabricated with putty type rubber impression material (Figure 3). After a fit check, the bonding of six anterior teeth is first achieved with a chemical cure adhesive. The posterior splinting sections are slipped onto the guide wires. During bonding, they are simply rotated into place while still on the guide wire. This technique simplifies the placement of the posterior sections.

### Patient 1

**Figure 4:**
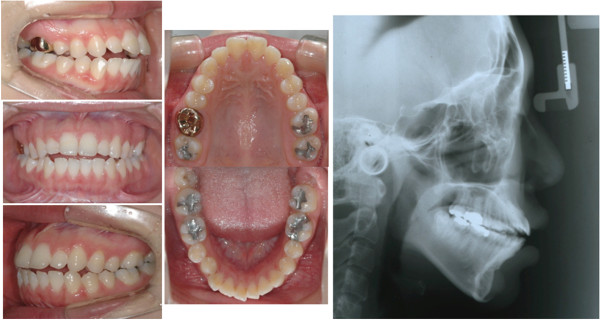
Initial records of case 1.

A 22-year-old female presented with a chief complaint of lip protrusion. She had Class I molar, Class II canine relationship, transverse discrepancy and anterior openbite. The lateral cephalogram revealed a skeletal Class II relationship, hyperdivergent pattern, and slight labioversion of upper and lower anterior teeth (Figure 4). She had CO-CR discrepancy without any subjective symptoms. After consultation with the TMD specialist, further splint or physical therapy was not indicated. The orthodontic treatment plan included extraction of the four first premolars and retraction with absolute anchorage. In order not to aggravate the vertical skeletal pattern, the KILBON system was chosen on the upper arch.

**Figure 5:**
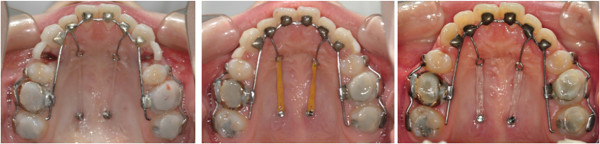
**Treatment progress on occlusal view of case 1.** Immediate after bonding, after 8 months, and 12 months.

The KILBON appliance was applied, and two TSADs were installed in paramedian area. She had minimal anterior crowding therefore, immediate space closure was initiated. For retraction, approximately 350 g of force was applied on each side with elastic chain connecting the anterior lingual retraction hook to the TSADs. The amount of retraction could be monitored by the length of the sliding wire protruding distally to the tube of the 1^st^ molar (Figure 5). Reactivation was done after 4 weeks. Conventional full fixed appliances were used for lower treatment.

### Patient 2

**Figure 6:**
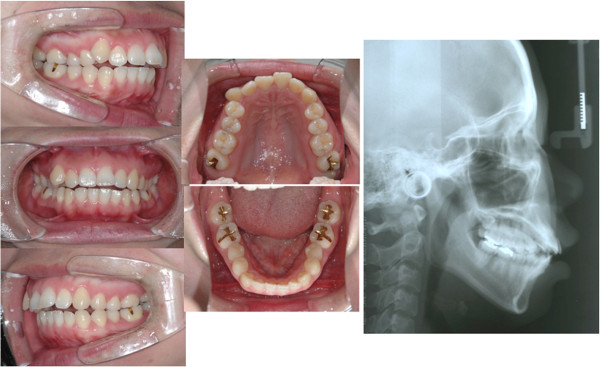
Initial records of case 2.

**Figure 7:**
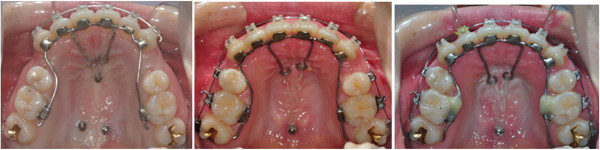
**Treatment progress on occlusal view of case 2.** Immediately after bonding, after 6 months, and after 10 months.

A 25-year-old female exhibited similar skeletal and dental problem with case 1 (Figure 6). She wanted conventional orthodontic treatment with labial appliances. For torque control of anterior teeth and total intrusion of maxillary arch, the KILBON system was combined during space closure. She showed crowding before treatment, We applied labial brackets for initial decrowding shortly. She had low palatal vault which restricts the lever arm length, therefore, the design of KILBON system was modified. Three TSADs were installed (two in paramedian area and one in the deepest point of the palatal rugae). The anterior TSAD was used for vertical anchorage before retraction. She wanted more retraction of upper lip after space closure, so the molar tube was splinted to the guide wire by composite resin and the upper dentition was further distalized (Figure 7).

## Results

### Patient 1

**Figure 8:**
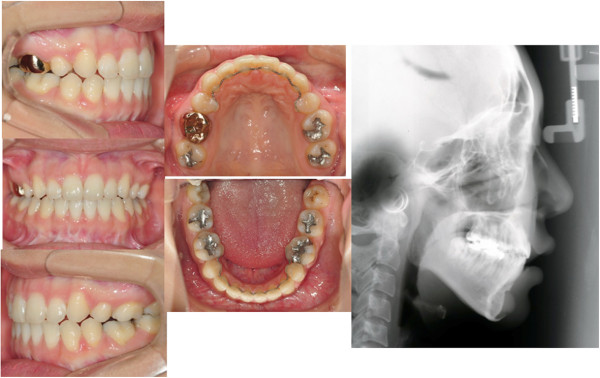
Final records of case 1.

**Figure 9:**
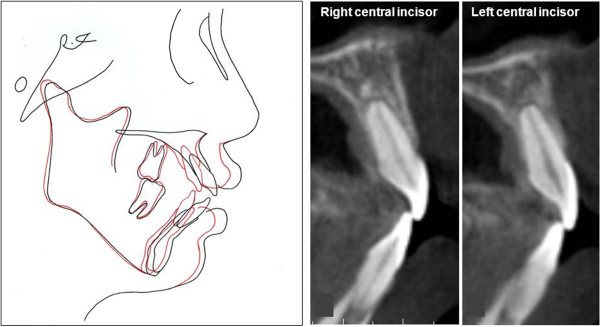
**Superimposition and sagittal image of upper central incisors after debonding using CBCT of case 1.** Both of them were well maintained within the alveolar housing.

After total 17 months of treatment, anterior protrusion was resolved and adequate overjet, and overbite, and stable occlusion were achieved (Figure 8). The maxillary anterior teeth experienced controlled tipping, which consisted of 4 mm intrusion and 7 mm retraction of the incisal edge. In spite of the large amount of retraction, there was no vertical alveolar bone loss or dehiscence on the incisors. The overall amount of intrusion of the upper molars was 1.5 mm. However, no autorotation was observed due to the repositioning of the mandible by the resolution of the CO-CR discrepancy (Figure 9).

### Patient 2

**Figure 10:**
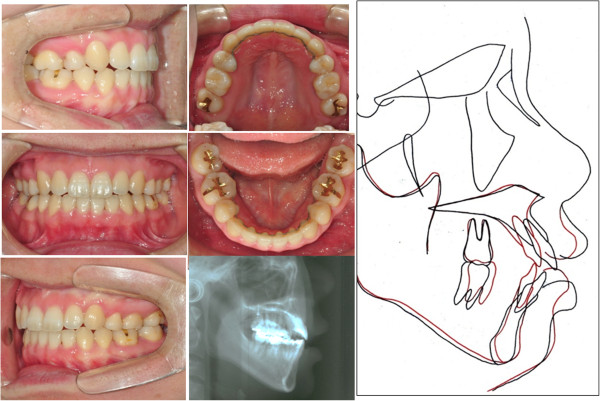
Final intraoral photographs, lateral cephalogram, and superimposition of case 2.

After 16 months of treatment periods, the maxillary anterior teeth showed bodily movement and the maxillary molars were intruded up to 1.5 mm, which was followed by autorotation of the mandible with 1.6 mm advancement of Pogonion (Figure 10).

## Discussion

Skeletal Class II hyperdivergent malocclusion has always been a challenge in orthodontics because of the sagittal and vertical discrepancies. Ye R et al. [9] reported that the hyperdivergent skeletal Class II malocclusion in non-growing patients has a steeper cant of the occlusal plane, and an excessive height of the maxillary incisors. Successful treatment requires flattening of occlusal plane by intrusion of anterior teeth and avoiding any increase in vertical height of the molar [10]. Conventional orthodontic treatment is likely to extrude the molars and rotate the mandible clockwise [11,12]. On the other hand, the KILBON system achieved total intrusion of maxillary dentition using only two mini-implants.

The KILBON system produced a large amount of intrusion and retraction of the anterior teeth, in a relatively short duration. Previous CBCT study after retraction of anterior teeth by conventional orthodontic treatment revealed dehiscence of palatal bone and vertical alveolar bone loss due to insufficient alveolar remodeling [13]. In our cases, alveolar bone remodeling, especially retraction of A point and the preservation of alveolar bone volume on pressure side, was achieved successfully. The teeth are grouped into three segments, so the orthodontic force is not concentrated on any individual tooth. Moreover, friction is minimal because the only site of friction during the sliding movement is between the posterior extension wire and the tube from the first molar. Labial flaring and round tripping of the incisors does not happen because the anterior teeth are not leveled and aligned before retraction. We do control the tube slot/guide wire using cephalogram and dental cast manually. Nowadays, Digital KILBON system was developed and we decide the exact tube slot position and angulation precisely using CAD/CAM method (Orapix, Seoul, Korea) [14].

The KILBON system can be further modified for effective vector control. If the patient has a low palatal vault, preliminary vertical force can be applied to the anterior teeth before retraction using an additional TSAD on the palatal rugae. Then the vertical height of the anterior teeth can be maintained by passive ligation between the TSAD and the lever arm during retraction. The clinician can also control the posterior intrusion with elastic modules between the molar splinting and the TSAD.

## Conclusions

This report presented an esthetic antero-posterior lingual sliding retraction system that provides simple and effective vertical and sagittal control of both the anterior and posterior teeth using palatal TSADs (KILBON System). The biomechanics are excellent and dependable for correcting a dentoalveolar protrusion in a patient with a Class II hyperdivergent skeletal pattern.

## Ethical approval

Written informed consent was obtained from all patients for publication of this report and the accompanying images. A copy of the written consent is available for review by the Editor-in-Chief of this journal. In all cases, a medical indication for the respective treatment was present. The surgical procedure constitutes a routine treatment. The authors declare that no ethical approval was necessary.

## Competing interests

We do not have any of the competing interests except Soon-Yong Kwon who holds the patent of this appliance but did not receive any reimbursements, fees, funding, or salary from an organization.

## Authors’ contributions

KS invented the appliance and treated patients using it. AH participated in writing the manuscript and consulted the result of the appliance effect. KS participated in invention and modification of the appliance. PY supervised the study design and the manuscript writing. CK participated in designing and coordination of this study. PC also participated in designing and coordination of the study. NG approved the final version of the study to be published. All authors read and approved the final manuscript.
